# Histopathological features and eight-week split-tablet sofosbuvir/velpatasvir treatment in young children with acute hepatitis C: a case-series study

**DOI:** 10.1186/s12879-026-12681-4

**Published:** 2026-01-30

**Authors:** Ya-Jie Pan, Ru-Yue Chen, Yan-Qin Ai, Qing-Lei Zeng

**Affiliations:** 1 Department of Infectious Diseases and Hepatology, The First Affiliated Hospital of Zhengzhou University, Zhengzhou, Henan Province China; 2 Department of Infectious Diseases, Xuchang Central Hospital, Xuchang, Henan Province China

**Keywords:** Acute, Children, Effectiveness, Hepatitis C virus, Sofosbuvir/velpatasvir

## Abstract

**Background and aims:**

Data on the clinical features and treatment of young children with acute hepatitis C (AHC) remain limited. This case-series report describes the clinical characteristics, histopathological findings, and treatment responses of two young children with AHC, aiming to provide insights for future research and clinical practice.

**Case summary:**

We evaluated two four-year-old children who acquired AHC through unsafe injection practices. The clinical decision-making process and management strategies were documented, and both children underwent liver biopsy. They received an eight-week course of sofosbuvir/velpatasvir (200/50 mg; ½ tablet, tablet-split, once daily). The primary efficacy endpoint was sustained virologic response (SVR12), defined as undetectable HCV RNA 12 weeks after completing treatment. Adverse events were monitored throughout the therapy period. Both children were asymptomatic at diagnosis. Baseline HCV RNA levels were 1,500 IU/mL and 2,300 IU/mL, with alanine aminotransferase (ALT) levels of 316 U/L and 15 U/L, respectively. Liver biopsy revealed mild inflammation with moderate fibrosis (G1S2) in child 1 and moderate inflammation with mild fibrosis (G2S1) in child 2. By the first week of therapy, ALT levels normalized in both cases; HCV RNA declined to 49.2 IU/mL in child 1 and became undetectable in child 2. By week 2, HCV RNA was undetectable in child 1. At 12 weeks after treatment completion, both achieved SVR12, with no treatment-related adverse events and stable laboratory parameters throughout.

**Conclusions:**

To our knowledge, this is the first report clearly demonstrating that AHC in young children can cause histological liver injury. An eight-week tablet-split regimen of sofosbuvir/velpatasvir appeared safe, well tolerated, and effective in these two cases. Further studies are warranted to validate these findings in the broader pediatric population.

## Introduction

Hepatitis C virus (HCV) infection is a global public health concern and a major risk factor for cirrhosis and hepatocellular carcinoma [1, 2]. According to the World Health Organization’s Global Hepatitis Report 2024, approximately 50 million people worldwide are living with chronic HCV infection [3]. Most infections are diagnosed at the chronic stage, as acute hepatitis C (AHC) is often asymptomatic or presents with only mild symptoms, resulting in frequent underdiagnosis or missed diagnosis. In 2021, the global incidence of AHC exceeded 7 million cases [4], with age-standardized rates similar between males and females and the highest incidence observed in children under 5 years of age [4]. Despite this, clinical and therapeutic research on AHC in children under 5 remains limited.

Between 2023 and 2024, a public health incident involving a cluster of HCV infections occurred at a clinic in Xuchang City, Henan Province, China. Dozens of individuals were diagnosed with acute and chronic HCV infection, including two children under five years of age (4.8 and 4.6 years, respectively) who had AHC. These cases were officially confirmed by the local Center for Disease Control and Prevention (CDC). An epidemiological investigation by the CDC revealed that the two children had no other identifiable risk exposures and had received only injections at this clinic for febrile illness, three and four months prior to their AHC diagnosis, respectively. Both children were asymptomatic at the time and displayed no clinical signs of illness; they were recalled for screening because other individuals in the same cluster had developed symptoms. Importantly, all household and close contacts—including parents, grandparents, siblings, as well as kindergarten teachers and classmates—tested negative for HCV, supporting the likelihood of a non-familial source of infection.

While the local government oversaw diagnosis and treatment of the affected patients, uncertainty remained regarding the management of these acute pediatric cases. Consequently, the children were referred to The First Affiliated Hospital of Zhengzhou University for further evaluation and treatment. This study provides a preliminary exploration through a detailed investigation of these two pediatric AHC cases, with the aim of informing future large-scale investigations. The study was reported in accordance with the CARE reporting guidelines [5].

## Patient characteristics, clinical decisions, and treatment outcomes

### Child 1

Child 1 was admitted on July 24, 2024. At the time of admission, the parents appeared highly anxious, likely due to the recent public health incident, and requested the most thorough diagnostic evaluation and optimal treatment. Upon admission, a comprehensive assessment of the patient’s general condition was conducted, including a detailed physical examination, virological and genotypic testing, biochemical analyses, and evaluation for other potential causes of liver injury.

The evaluation revealed that child 1 tested negative for hepatitis A virus, hepatitis B virus, human immunodeficiency virus, Epstein-Barr virus, and cytomegalovirus. Notably, his HCV RNA level was 1,500 IU/mL, and alanine aminotransferase (ALT) was elevated at 316 U/L (Table 1). Although alkaline phosphatase was elevated, this was considered likely related to normal growth and development. Serum bilirubin, renal function, blood lipids, and blood glucose were all within normal ranges. HCV genotyping confirmed infection with genotype 1b.

**Table 1:** Baseline characteristics

Characteristics	Child 1	Child 2
Gender	Male	Male
Age, years	4.8	4.6
Height, cm	112	115
Weight, kg	19	21.6
Transmission route	Unsafe injection	Unsafe injection
HCV RNA, IU/mL^*^	1500	2300
HCV genotype^**^	1b	1b
**Liver function**		
Alanine aminotransferase (Normal range < 40 U/L)	316	15
Aspartate aminotransferase (Normal range < 40 U/L)	147	18
Alkaline phosphatase (Normal range < 125 U/L)	254	141
Gamma-glutamyl transpeptidase (Normal range < 58 U/L)	23	8
Total bilirubin (Normal range < 17.1 µmol/L)	11.3	5.6
Albumin (Normal range 35–55 g/L)	45.2	48.9
Prothrombin time activity (Normal range 70–150%)	86	92
**Abdominal ultrasound**	Splenomegaly	Normal

Abbreviation: HCV, hepatitis C virus. ^*^Serum HCV RNA was monitored using the Roche COBAS^®^ AmpliPrep/COBAS^®^ TaqMan^®^ HCV Test (Roche Molecular Systems; cutoff value, 15 IU/mL). ^**^HCV genotype was determined via gene sequencing assay

However, it remained unclear whether antiviral treatment was necessary. This uncertainty was driven by multiple factors, including the potential for spontaneous HCV clearance in children, the lack of established treatment guidelines for AHC in patients under 6 years of age, the absence of approved pediatric formulations of direct-acting antivirals (DAAs) in China, limited data on their safety and efficacy in this population, and the obsolescence of interferon-based regimens despite our previous clinical experience [6].

At the same time, several considerations supported intervention. The probability of spontaneous HCV clearance was relatively low, and the risk of progression to chronic infection was substantial. Although no established treatment recommendations exist for AHC in children aged 3–6 years, antiviral therapy is available for chronic hepatitis C in children older than 3 years [1]. Moreover, viral hepatitis-related stigma and discrimination remain serious issues in Chinese society [7], further emphasizing the potential benefit of early treatment.

Beyond the clinical considerations, we also faced substantial pressure from the patient’s family, who strongly requested active treatment with the expectation of cure. In addition to their profound fear of the disease, the parents expressed concerns about potential stigma and discrimination once the child returned to kindergarten. During the public health investigation, the child’s HCV status had become known within the kindergarten and the surrounding community.

At that stage, the medical team remained cautious about initiating antiviral therapy. We also explained that HCV is not transmitted through casual daily contact. Although the parents acknowledged this information, they remained concerned that other parents at the kindergarten and nearby neighbors might not share this understanding, potentially resulting in ongoing stigma and discrimination. After multiple multidisciplinary discussions, a decision was made to perform a liver biopsy. This decision was based on the observation that ALT levels exceeding 300 U/L are relatively uncommon in hepatitis C in routine clinical practice. In addition, the possibility of concomitant liver diseases that are difficult to distinguish through hematological evaluation could not be entirely excluded. Accordingly, after written informed consent from the child’s parents, a liver biopsy was performed to assess histopathological features and disease severity, and to guide subsequent treatment decisions.

**Fig. 1:**
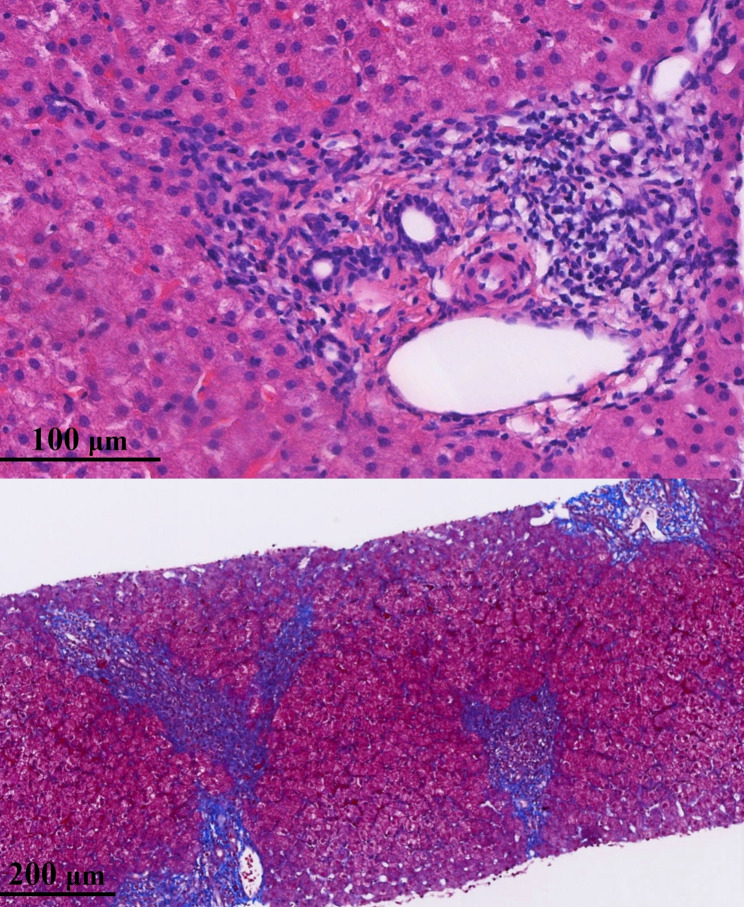
Histopathological findings of the liver in child 1. Upper: Histological examination revealed marked infiltration of inflammatory cells, predominantly lymphocytes, in the portal areas (G1, Hematoxylin–eosin staining). Lower: Portal fibrosis was evident, with fibrous tissue extending into adjacent regions. Fibrous septa were present between hepatic lobules, but no bridging fibrosis was observed (S2, Masson’s trichrome staining)

Histological evaluation revealed mild liver inflammation with moderate fibrosis in child 1 (G1S2, Fig. 1), and no other potential causes of liver disease were identified. Hepatic inflammation and fibrosis were graded and staged according to the modified histological activity index (HAI) described by Scheuer [8]. This histopathological finding was unexpected. Following consultation with the Department of Pathology, the diagnosis was independently confirmed by three senior pathologists. These results further heightened the parents’ anxiety and distress. At this point, the clinical team began to favor initiation of antiviral therapy.

After repeated discussions within the medical team, we decided to initiate treatment using an eight-week regimen of half-dose sofosbuvir/velpatasvir (200/50 mg; ½ tablet, tablet-split, one bottle of Epclusa^®^). We explained this treatment regimen and the subsequent monitoring plan to the patient’s family. The decision to use tablet-splitting was driven by the absence of pediatric formulations, our prior experience curing a 1.2-year-old child with chronic hepatitis C using tablet-splitting [9], previous experience with ribavirin tablet-splitting [6], and considerations of reducing medical costs. The choice of an eight-week treatment course was based on the patient’s acute infection with a low viral load. According to the 2023 American hepatitis C guidance, an eight-week regimen is recommended for genotype 1 patients without cirrhosis, applicable to those who have an HCV RNA level < 6 million IU/mL [1]. We communicated with the patient’s family that the primary efficacy endpoint would be undetectable HCV RNA 12 weeks after treatment completion (SVR12), which would be considered indicative of cure.

During therapy, patients underwent weekly monitoring until achievement of undetectable HCV RNA and normalization of ALT levels, together with routine biochemical assessments. Further evaluations were performed at the end of treatment (week 8) and at 12 weeks after treatment cessation, both of which included abdominal ultrasonography. After one week of treatment (Table 2), child 1’s HCV RNA level had decreased to 49.2 IU/mL, with normalization of ALT. By the second week (Table 3), HCV RNA was undetectable. The child was then discharged and continued to be closely monitored as an outpatient. At the end of the eight-week treatment course (Table 4), child 1 maintained normal ALT levels and undetectable HCV RNA, ultimately achieving SVR12 (Table 5). No specific adverse events were observed during treatment or follow-up. At SVR24, child 1’s spleen had returned to normal size.

**Table 2:** Clinical testing results at week 1 of treatment

Characteristics	Child 1	Child 2
**HCV RNA**,** IU/mL**	49.2	undetectable
**Liver function**		
Alanine aminotransferase (Normal range < 40 U/L)	26	18
Aspartate aminotransferase (Normal range < 40 U/L)	27	23
Alkaline phosphatase (Normal range < 125 U/L)	141	136
Gamma-glutamyl transpeptidase (Normal range < 58 U/L)	16	11
Total bilirubin (Normal range < 17.1 µmol/L)	8.3	5.0
Albumin (Normal range 35–55 g/L)	46.6	47.0

Abbreviation: HCV, hepatitis C virus

**Table 3:** Clinical testing results at week 2 of treatment

Characteristics	Child 1	Child 2
**HCV RNA**,** IU/mL**	undetectable	-
**Liver function**		
Alanine aminotransferase (Normal range < 40 U/L)	14	-
Aspartate aminotransferase (Normal range < 40 U/L)	27	-
Alkaline phosphatase (Normal range < 125 U/L)	162	-
Gamma-glutamyl transpeptidase (Normal range < 58 U/L)	12	-
Total bilirubin (Normal range < 17.1 µmol/L)	7.5	-
Albumin (Normal range 35–55 g/L)	48.1	-

Abbreviation: HCV, hepatitis C virus

**Table 4:** Clinical testing results at week 8 of treatment

Characteristics	Child 1	Child 2
**HCV RNA**,** IU/mL**	undetectable	undetectable
**Liver function**		
Alanine aminotransferase (Normal range < 40 U/L)	15	15
Aspartate aminotransferase (Normal range < 40 U/L)	29	17
Alkaline phosphatase (Normal range < 125 U/L)	177	141
Gamma-glutamyl transpeptidase (Normal range < 58 U/L)	7	8
Total bilirubin (Normal range < 17.1 µmol/L)	6.2	5.6
Albumin (Normal range 35–55 g/L)	49.2	48.9

Abbreviation: HCV, hepatitis C virus

**Table 5:** Clinical testing results at posttreatment week 12

Characteristics	Child 1	Child 2
**HCV RNA**,** IU/mL**	undetectable	undetectable
**Liver function**		
Alanine aminotransferase (Normal range < 40 U/L)	12	17
Aspartate aminotransferase (Normal range < 40 U/L)	28	20
Alkaline phosphatase (Normal range < 125 U/L)	203	157
Gamma-glutamyl transpeptidase (Normal range < 58 U/L)	10	7
Total bilirubin (Normal range < 17.1 µmol/L)	9.6	6.4
Albumin (Normal range 35–55 g/L)	49.7	50.9
**Abdominal ultrasound**	Splenomegaly^*^	Normal

Abbreviation: HCV, hepatitis C virus. ^*^At 24 weeks after treatment completion, Child 1’s spleen had returned to normal size

### Child 2

Initially, we were unaware of the existence of child 2. Child 2 was admitted on August 1, 2024, eight days after child 1. The two children originated from the same town and attended the same kindergarten class, and they were placed in the same hospital room after admission. At the time of child 2’s admission, child 1 had already completed all evaluations, including pathological examination, and had started the second day of sofosbuvir/velpatasvir treatment.

Like child 1’s parents, child 2’s parents exhibited extreme anxiety. However, one notable difference was that they strongly requested that the treatment follow exactly the same approach as child 1. Through communication, we learned that, from their perspective, if child 1 were cured, child 2 would be the only child with hepatitis C left in the class, the town, and the surrounding area. Since everyone around them was aware, they believed child 2 would inevitably face discrimination. Accordingly, we proceeded with the same protocol.

**Fig. 2:**
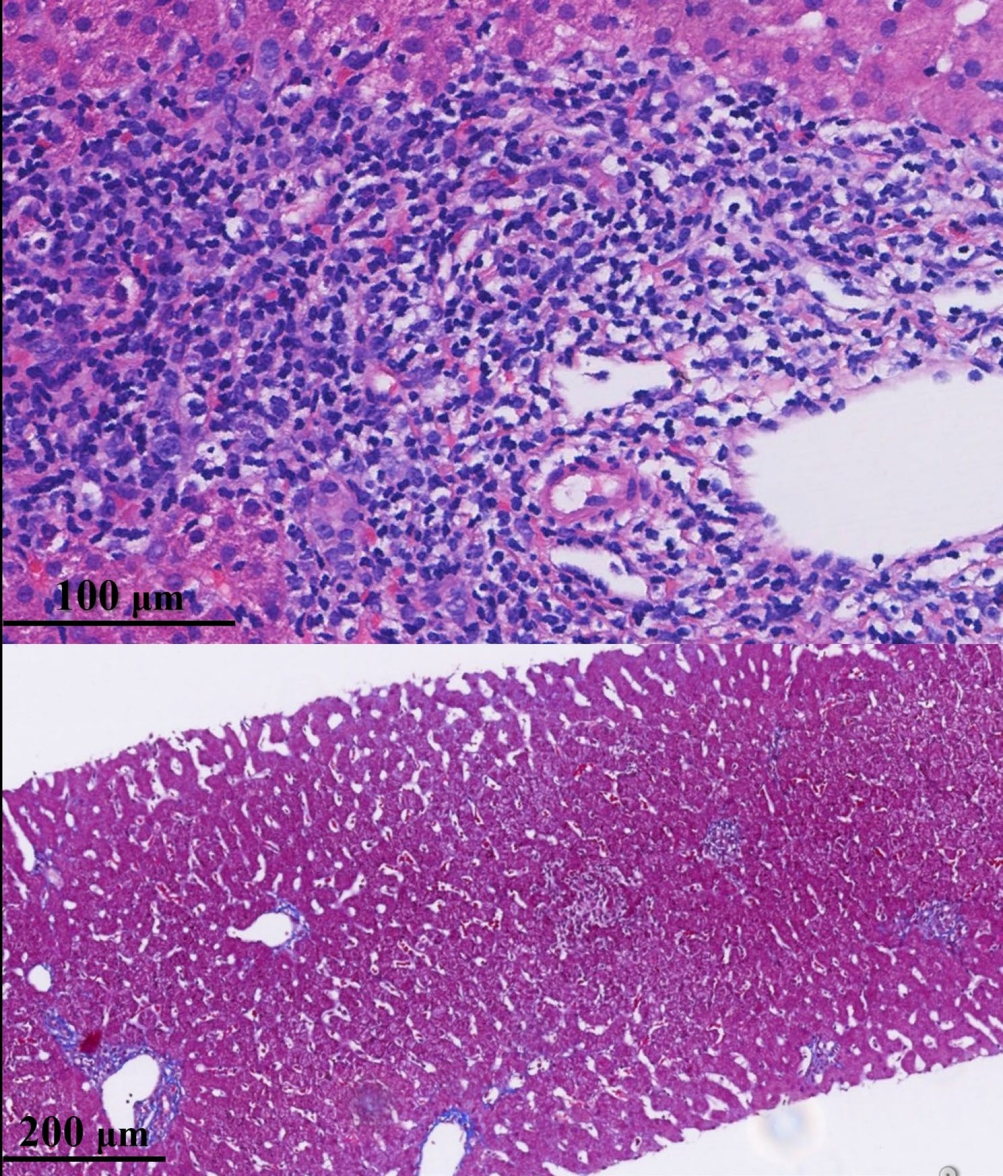
Histopathological findings of the liver in child 2. Upper: Histological examination revealed more pronounced infiltration of inflammatory cells, predominantly lymphocytes, in the portal areas (G2, Hematoxylin–eosin staining). Lower: Fibrous tissue was limited in distribution, primarily confined to the portal areas, without significant extension into the hepatic lobules or formation of fibrous septa (S1, Masson’s trichrome staining)

Upon evaluation, he tested negative for hepatitis A virus, hepatitis B virus, human immunodeficiency virus, Epstein-Barr virus, and cytomegalovirus. Notably, his HCV RNA level was 2,300 IU/mL, with an ALT level of 15 U/L (Table 1). Although alkaline phosphatase was also elevated, this was considered likely related to normal growth and development. Serum bilirubin, renal function, blood lipids, and blood glucose were all within normal ranges. HCV genotyping confirmed infection with genotype 1b. Histological evaluation revealed moderate hepatitis with mild fibrosis (G2S1; Fig. 2). This histopathological finding was also unexpected, as it occurred despite normal ALT levels in the child.

After one week of treatment (Table 2), child 2 achieved undetectable HCV RNA and maintained ALT normalization. The child was then discharged and continued to be monitored as an outpatient. Child 2’s hospital stay was approximately one week shorter than that of child 1. At the end of the eight-week treatment course (Table 4), child 2 maintained normal ALT levels and undetectable HCV RNA, ultimately achieving SVR12 (Table 5). No specific adverse events were observed during treatment or follow-up.

## Discussion

The incidence of AHC is increasing, having risen by 124% in the United States between 2013 and 2020 [1]. Treating these populations is therefore essential for both the prevention and eventual elimination of HCV. Although both children were diagnosed with acute HCV infection, initiating antiviral therapy was initially uncertain due to their young age, absence of symptoms, and lack of established treatment guidelines for pediatric AHC. Liver biopsy findings, however, revealed varying degrees of hepatic inflammation and fibrosis, yielding unexpected results that indicated ongoing liver injury despite the absence of clinical symptoms. Given the risk of disease progression, the psychosocial burden of prolonged HCV monitoring in children, and the potential for discrimination, early intervention was deemed appropriate.

Actually, the clinical data on AHC in young children are limited, with most reports dating back to the era before the HCV was formally identified and named—when it was referred to as non-A, non-B hepatitis [10, 11]. The causal relationships in those early reports were often unclear. Since the identification of HCV, AHC, especially in young children, has become difficult to detect in a timely manner. In this study, the exposure time, testing time, and diagnosis time were clearly documented, confirming that both pediatric cases were infected for less than six months.

Regarding the histopathological features of hepatitis C in young children, we previously reported chronic cases approximately a decade ago and found that patients with G0S0 were rare [6]. Among 163 HCV RNA–positive children with chronic hepatitis C and a median age of 2.6 years, 23.9% exhibited grade G2 inflammation activity, and 29.5% exhibited stage S2 liver fibrosis [6]. However, at that time, the distinct pathological characteristics of AHC remained unclear. This study provides a clear and direct depiction of the histopathological features of AHC in two young children. The findings challenge the traditional belief that HCV infection requires decades to induce liver fibrosis [12]. The mechanisms underlying early fibrotic changes are uncertain but may involve rapid activation of hepatic stellate cells triggered by virus-induced inflammation and immune-mediated hepatocellular injury [13, 14]. Pre-existing liver disease was excluded based on medical history and prior laboratory results. Biopsy samples were independently reviewed by three pediatric pathologists. While mild fibrosis is uncommon in acute pediatric HCV, our findings add to rare reports of early fibrogenesis in AHC children, suggesting that fibrosis can develop even during the initial phase of infection.

While previous studies have explored shortened six-week DAAs regimens for acute or recent HCV infection, the results have been suboptimal [1]. Specifically, trials involving ledipasvir/sofosbuvir [15], glecaprevir/pibrentasvir [16], and sofosbuvir/velpatasvir [17] reported lower SVR12 rates compared to standard-duration therapy. Consequently, current American guidance does not recommend abbreviated six-week DAAs courses for acute HCV infection [1]. Based on prior studies [18–20], the guidance currently recommends that all chronically HCV-infected children and adolescents aged ≥ 3 years be treated with an approved DAAs regimen, regardless of disease severity [1]. The recommended treatment duration for sofosbuvir/velpatasvir is 12 weeks [1]. However, to date, no studies have specifically evaluated the use of sofosbuvir/velpatasvir in young children with AHC.

Due to the absence of pediatric formulations in China and building upon our prior clinical experience in managing very young child with chronic HCV infection [9], sofosbuvir/velpatasvir (200/50 mg) tablets were split to achieve the recommended pediatric dose in accordance with the drug label. We ultimately opted for an eight-week regimen of half-dose sofosbuvir/velpatasvir (exactly one bottle of Epclusa^®^), tailored to the children’s body weight, achieving favorable outcomes without adverse events. As mentioned, the main reason for selecting an eight-week treatment course was the patients’ acute infection with a low viral load. Additionally, current guidance recommends an eight-week regimen for genotype 1 patients without cirrhosis [1], and economic considerations played a minor role, as one bottle of medication via tablet-splitting sufficed for an eight-week course. This approach aligns with previous study in adults with AHC treated with an eight-week course of sofosbuvir/velpatasvir [21]. Taken together, the results of this case series suggest that an eight-week regimen may represent a potential option for determining treatment duration in future clinical trials of pediatric AHC.

Several limitations of this study should be acknowledged. First, spontaneous viral clearance is possible in acute HCV infection [22, 23]; however, the decision to initiate treatment also took into account the parents’ strong preference for therapy, the time and economic costs associated with prolonged monitoring, the psychological burden during follow-up, and the potential risk of social discrimination. Second, although we previously applied a split-tablet regimen in a 1.2-year-old child with chronic hepatitis C [9], the pharmacokinetics of split-tablet dosing in very young children remain uncertain, and this approach was adopted out of necessity due to the lack of approved pediatric formulations. Third, histopathological evaluation was conducted at a single center, which may introduce interpretative bias; however, the biopsy specimens were independently reviewed by three experts with concordant results, and the severity assessment was limited to staging of inflammation and fibrosis (G/S grading), which represent relatively straightforward components of liver pathology. Finally, the cases originated from an iatrogenic cluster, which may limit generalizability to sporadic pediatric acute HCV infections. Nevertheless, these unique cases provide valuable real-world insights into early intervention strategies for pediatric acute HCV in settings where approved pediatric formulations are unavailable.

In conclusion, this study demonstrates that young children with AHC can exhibit significant liver injury and suggests that a shortened eight-week course of sofosbuvir/velpatasvir may represent a promising treatment option in pediatric AHC. Future larger prospective studies are needed to confirm the safety and efficacy of this approach.

## Data Availability

The authors confirm that the data supporting the findings of this study are available in this article. All datasets developed for this study are available from the corresponding author (Q.-L.Z.) upon reasonable request via e-mail.

## Abbreviations

AHC: Acute hepatitis C
ALT: Alanine aminotransferase
CDC: Center for Disease Control and Prevention
DAAs: Direct-acting antivirals
HCV: Hepatitis C virus
SVR: Sustained virological response
